# Development and Validation of a Sub-National, Satellite-Based Land-Use Regression Model for Annual Nitrogen Dioxide Concentrations in North-Western China

**DOI:** 10.3390/ijerph182412887

**Published:** 2021-12-07

**Authors:** Igor Popovic, Ricardo J. Soares Magalhães, Shukun Yang, Yurong Yang, Erjia Ge, Boyi Yang, Guanghui Dong, Xiaolin Wei, Guy B. Marks, Luke D. Knibbs

**Affiliations:** 1 Faculty of Medicine, School of Public Health, University of Queensland, Herston 4006, Australia; 2 UQ Spatial Epidemiology Laboratory, School of Veterinary Science, University of Queensland, Gatton 4343, Australia; r.magalhaes@uq.edu.au; 3 Children’s Health and Environment Program, UQ Children’s Health Research Center, The University of Queensland, South Brisbane 4101, Australia; 4 Department of Radiology, The Second Affiliated Hospital of Ningxia Medical University, The First People’s Hospital in Yinchuan, Yinchuan 750004, China; shukunyang@163.com; 5 Department of Pathogenic Biology & Medical Immunology, School of Basic Medical Science, Ningxia Medical University, Yinchuan 750004, China; yangyurong@hotmail.com; 6 Dalla Lana School of Public Health, University of Toronto, Toronto, ON M5S 1A1, Canada; erjia.ge@utoronto.ca (E.G.); xiaolin.wei@utoronto.ca (X.W.); 7 Guangdong Provincial Engineering Technology Research Center of Environmental Pollution and Health Risk Assessment, Department of Occupational and Environmental Health, School of Public Health, Sun Yat-sen University, Guangzhou 510085, China; yangby23@mail.sysu.edu.cn; 8 Guangzhou Key Laboratory of Environmental Pollution and Health Risk Assessment, Department of Preventive Medicine, School of Public Health, Sun Yat-sen University, Guangzhou 510085, China; donggh5@mail.sysu.edu.cn; 9 South Western Sydney Clinical School, University of New South Wales, Liverpool 2170, Australia; g.marks@unsw.edu.au; 10 Woolcock Institute of Medical Research, Glebe 2037, Australia; 11 Centre for Air Pollution, Energy and Health Research, Glebe 2037, Australia; luke.knibbs@sydney.edu.au; 12 Faculty of Medicine and Health, School of Public Health, The University of Sydney, Camperdown 2006, Australia

**Keywords:** air pollution modelling, nitrogen dioxide, satellite-based model, land-use regression, exposure assessment, China

## Abstract

Existing national- or continental-scale models of nitrogen dioxide (NO_2_) exposure have a limited capacity to capture subnational spatial variability in sparsely-populated parts of the world where NO_2_ sources may vary. To test and validate our approach, we developed a land-use regression (LUR) model for NO_2_ for Ningxia Hui Autonomous Region (NHAR) and surrounding areas, a small rural province in north-western China. Using hourly NO_2_ measurements from 105 continuous monitoring sites in 2019, a supervised, forward addition, linear regression approach was adopted to develop the model, assessing 270 potential predictor variables, including tropospheric NO_2_, optically measured by the Aura satellite. The final model was cross-validated (5-fold cross validation), and its historical performance (back to 2014) assessed using 41 independent monitoring sites not used for model development. The final model captured 63% of annual NO_2_ in NHAR (RMSE: 6 ppb (21% of the mean of all monitoring sites)) and contiguous parts of Inner Mongolia, Gansu, and Shaanxi Provinces. Cross-validation and independent evaluation against historical data yielded adjusted R^2^ values that were 1% and 10% lower than the model development values, respectively, with comparable RMSE. The findings suggest that a parsimonious, satellite-based LUR model is robust and can be used to capture spatial contrasts in annual NO_2_ in the relatively sparsely-populated areas in NHAR and neighbouring provinces.

## 1. Introduction

Air pollution is implicated in an estimated 4 million premature deaths globally each year [1]. The increasing global health burden attributable to air pollution is driven by population and economic growth, reflecting rapid industrialisation and urbanization, particularly in developing nations [2,3]. The highest burden of disease due to outdoor air pollutants as quantified by Disability Adjusted Life Years (DALYs) or years lost due to ill-health has consistently been recorded in Asia and Africa (2029–2751 age-standardised DALYs per 100,000 population) [4,5]. Similarly, global death rates (age-standardised) attributable to ambient air pollution vary by a factor of 10 between high-income countries (8 per 100,000 population) and low-to-middle-income countries (LMICs, 85 per 100,000 population) [1]. These severe health inequalities highlight the need for valid estimates of air pollution exposure in LMICs.

Of the major outdoor air pollutants, nitrogen dioxide (NO_2_) is one of the key indicators of anthropogenic sources, such as traffic and industrial emissions [6,7]. NO_2_ is also an important precursor of other important air pollutants, such as ground-level ozone. However, the spatially heterogenous nature and diverse range of NO_2_ sources presents several challenges for modelling [8,9]. For instance, NO_2_ levels have been shown to decay rapidly within ~150 to 200 m of major roads [6,9]. Industrial activities such as combustion of fossil fuels in power stations can affect NO_2_ concentrations more subtly but over greater distances [10]. Generally, in urban settings, NO_2_ sources include coal combustion and vehicle emissions, whilst in less populated areas, contributors to NO_2_ levels are more likely to be regional-scale industrial emissions [11,12].

The rural and urban contrast in terms of NO_2_ sources is particularly apparent in China. At the start of this century, environmental policies in growing urban centres, such as Beijing and Shanghai, have led to a transition from coal to cleaner energy sources. There have also been restrictions on high-emitting vehicles [12,13]. In rural areas, however, coal is still widely used for power generation and at household level for cooking and heating. In farming areas, the use of agricultural fertilisers, machinery, and equipment can also affect NO_2_ concentrations [14]. As a result, NO_2_ exposure assessment requires consideration of both local and regional sources of the pollutant. This is particularly relevant to China, which has distinctively different sources of NO_2_ in large cities compared with sparsely populated rural areas in the north-west of the country [12,14].

Land-use regression (LUR) is an approach to NO_2_ exposure assessment modelling that has been used since the mid 1990s. More recently, however, LUR models have sought to combine satellite observations and ground level measurements with known predictors of NO_2_ pollution [15,16]. An LUR model offers improved spatial coverage and discrimination compared with what is achievable with fixed monitoring stations alone, as it can be applied to estimate air pollution exposure even in non-measured locations at an intra-urban or local scale [17,18]. There has been increased research interest in satellite-based LUR modelling of NO_2_, where tropospheric NO_2_ and other remotely sensed variables are used as a spatially-varying predictor of measured ground-level concentrations. However, existing literature is limited to global, continental models or large urban centres [7,15,16,18,19,20].

Current LUR models in China have potential limitations regarding their coarse spatial resolution (>10 km) and temporal span, which may limit validity of estimates of fine-scale intra-urban NO_2_ concentrations [21,22,23]. Additionally, the relatively limited number of monitoring sites (sample size) included in model development in previous sub-national models for China may limit the validity of their NO_2_ predictions [21,23,24,25,26]. The use of less spatially refined (13 × 24 km^2^ at nadir) NO_2_ satellite measurements and other geographically varying predictors is also a limiting feature in current NO_2_ models in the country [21,23,24,25,26,27]. Whilst spatially refined and cross-validated global and national LUR models exist for China, their validity and generalisability to sparsely populated areas is unknown, as is the question of whether models with smaller spatial extent (e.g., sub-national) are valid in those areas [7,28].

As existing air pollution models have focused on understanding the spatial variability of NO_2_ in more densely inhabited local urban environments, and at a national scale, the ability of such models to assign exposure associated with rural emissions is unclear. Therefore, the objective of this study was to develop a LUR model for annual NO_2_ levels in Ningxia Hui Autonomous Region (NHAR) a sparsely populated province with income levels and life expectancy well below the average for China [29]. The development of the model was motivated by intention of examining the effects of NO_2_ and other ambient pollutants on respiratory disease outcomes in Ningxia Hui Autonomous Region. We aimed to capture important sources of NO_2_ exposure in a sparsely-populated region of China for which there are no comparable sub-national models. We also sought to independently validate the model against historical measurements. The findings presented in this study may inform future NO_2_ exposure assessment applications outside the study area by identifying important predictors of NO_2_ in other rural and semi-rural regions in China and beyond.

## 2. Materials and Methods

### 2.1. Study Area

Ningxia Hui Autonomous Region is the smallest provincial-level autonomous region located in Northwest China. Ningxia’s territory covers an area of 66,400 km^2^. Administratively, NHAR is divided into five prefecture level cities (Yinchuan (2.3 million population); Shizuishan (734,400 population); Wuzhong (1.4 million population); Guyuan (1.2 million population); and Zhongwei (1.2 million population)) that are subsequently subdivided into counties/districts/county-level cities, townships, and villages [29,30,31]. The total population of NHAR exceeds 7.2 million people (>46% of the population inhabit rural areas), ranking 30th and 28th in population size and density (108 persons per km^2^), respectively, of 34 provincial level administrative divisions in China [31]. NHAR has one of the lowest GDP outputs in the country [29,31].

**Figure 1:**
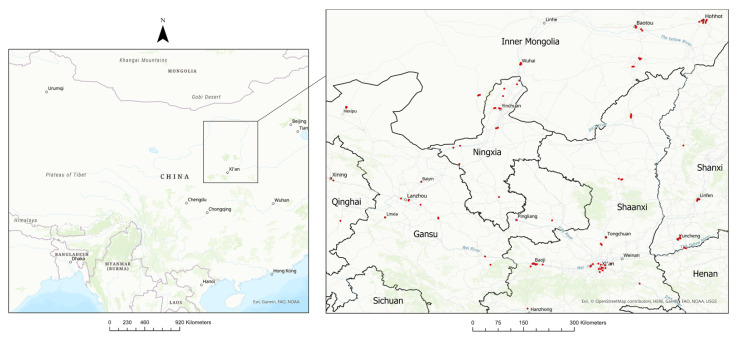
Location and distribution of air quality monitoring sites in study area.

The study area was extended to a 500 km radius centred on Ningxia province as a balance between increasing the number of monitoring stations without substantially changing the relationship between predictors and NO_2_ (i.e., the study area was assumed to be broadly similar to NHAR in NO_2_ sources and spatial variability). By expanding the study area beyond Ningxia, the number of available monitoring stations increased from 19 to 123 (Table S1). As a result, the study area also encompassed parts of Inner Mongolia Autonomous Region (24 million population), Gansu (25 million population), as well as Shaanxi Province (24 million population) (Figure 1). Including Shaanxi Province ensured inclusion of important regional sources and predictors of NO_2_ pollution, such as mining sites and coal power stations. Dominant sources of NO_2_ emissions are primarily located in south-eastern parts of Shaanxi, namely in Tongchuan and Xi’an as well as in Baoji. Shaanxi Province accounted for 34% of monitoring sites in the study region. Nitrogen oxide emitting iron ore processing sites located in western areas of Gansu Province (Lanzhou, Linxi, and Baiyin) were also captured in addition to petrochemical processing bases and refining industries found in north-east of Yinchuan in Ningxia Province [32].

#### 2.1.1. Measured NO_2_

Continuous, hourly ground-level NO_2_ measurements were obtained for the period of 1 January to 31 December 2019 through OpenAQ [33]. This year was the first year for which hourly NO_2_ measurements were publicly available for a full calendar year. OpenAQ is an open-source portal that aggregates government-measured ambient air pollution data and obtains air monitoring data for China from the Chinese Environmental Monitoring Center (CEMC) as well as provincial-level Environmental Monitoring Agencies [33]. All measurements were performed using standard chemiluminescence methods, following Chinese Ambient Air Quality Standards (GB 3095—2012) and Ambient Air Quality Index (AQI) technology (HJ 633—2012).

Quality control and consistency checks were applied to the daily monitoring data to exclude monitoring sites with incomplete data (i.e., where measurements were not available for at least 75% of the year) and/or missing coordinates (latitude and longitude to five decimal places). A total of 123 sites were identified within a 500 km radius centred on NHAR. Due to the strong seasonal variation in NO_2_, we sought to limit the potential for bias due to missing data, and 14 sites were therefore removed (>25% missing daily monitoring data in the year) [34]. Annual averages for 2019 were calculated for the remaining 109 sites.

#### 2.1.2. Variables

Satellite and non-satellite-based predictors of NO_2_, were extracted from various data sources (Table S2) and are summarised in Table 1. Selection of predictors that could best capture the spatial variability of NO_2_ was informed by previous global, continental, and national LUR models [7,15,17,19,28]. Predictors were calculated as averages within an area defined by a circle of a specified radius around the monitoring site location (“buffers” with 22 radii ranging from 100 m to 10 km) or point estimates for the monitoring site.

### 2.2. Model Development

#### 2.2.1. Variable Selection

A total of 270 predictors were included for initial model development for the year 2019, consisting of 264 buffer and six point variables. Predictors with >75% missing, repeating (i.e., identical), or zero values across the monitoring sites were excluded (n = 88), leaving 182 for model development [16]. The predictors were centred and standardised to aid interpretation and model convergence. The expected coefficient direction of each predictor variable, based on empirical knowledge, was pre-defined (Table S2) following standard LUR modelling practice [35,36]. Briefly, predictors reflecting sources of NO_2_ such as roads, impervious surfaces, and population density, were defined to be positively associated with NO_2_ levels. Predictors, such as water and tree and vegetation cover, were defined as being negatively associated with NO_2_.

#### 2.2.2. Model Development

A supervised forward stepwise linear regression approach was used. Starting with the variable most correlated with measured NO_2_, variables were added to the model if: (i) the regression coefficient followed the prespecified effect direction, (ii) they did not change the coefficient direction of a variable already in the model, and (iii) they increased adjusted R^2^ of the model by >1% [16,17,19]. The selection process was repeated until no variables remained that satisfied the inclusion criteria. Forward stepwise linear regression and diagnostic checks was performed using olsrr package in RStudio Version 1.4.1106 [37].

#### 2.2.3. Model Diagnostics and Cross-Validation

Residual plots were visually checked (Figure S1), while collinearity was assessed using variance inflation factor (VIF), with values >3 suggesting collinearity (Table 2). An examination of influential observations was conducted using Cook’s distance (>4/n) and df-beta values (>2/√n) (Figures S2 and S3). For all diagnostic metrics, any suspect sites were sequentially removed and their effect on model composition and performance evaluated. Each of these sites was also manually investigated in Google Earth to understand the nature of the site and inform decisions about retaining or excluding the monitoring site in the final model. For example, one of the sites was situated on a mountain peak, which due to its elevated location, may have been subject to greater dispersion and wind dilution. Concentrations captured by this site (7 ppb) were significantly lower than measurements recorded in other areas in the city of Tianshui (Gansu Province) (26–40 ppb). Moran’s *I* was used in ArcGIS to detect spatial autocorrelation in model residuals (Figure S4).

The prediction error in terms of root-mean-square error (RMSE (in ppb and also as a percentage of the mean of all monitoring sites)), mean absolute error (MAE), and R^2^ of the final model was evaluated using 5-fold cross-validation. This approach partitions the data into a training and a model building sub-set. The monitoring sites were randomly divided into five sub-sets using the caret package in RStudio Version 1.4.1106 [38]. Four data sub-sets were used to train the model and the remaining group to test the model developed. The cross-validation process was repeated between 50 and 500 times to assess stability of validation metrics (Table S3).

#### 2.2.4. Independent Evaluation

Independent evaluation of the final model was conducted using historical annual NO_2_ measurements for 2014 and 2015 from monitoring sites in the study area that were not available for developing the 2019 annual model (n = 41) (Table S4). Independent sites were identified using a previously published global scale NO_2_ model [19]. As with the development sites, the independent monitoring stations also used chemiluminescence to measure NO_2_ concentrations. The LUR model was applied to estimate the average yearly concentration for these sites (year 2014 = 16; year 2015 = 25) by matching annual time-varying predictor variables to the same period and using the 2019 regression coefficients [16]. The aim was to externally evaluate the LUR model’s performance using new measurements. Specifically, we assessed the validity of applying 2019 model coefficients up to five years earlier. The same validation metrics as cross-validation were used (Table S5).

#### 2.2.5. Model Predictions

Model predictions were gridded at 100 m × 100 m for visualisation of spatial patterns across the study area. NO_2_ estimates for each grid cell centroid were calculated by multiplying the year 2019 regression coefficients to the corresponding predictor values. Historical predictions (2005–2018) were obtained by matching annual time-varying predictor variables for each year and using the 2019 regression coefficients. Population-weighted annual average NO_2_ levels for the 358 township-level divisions across NHAR (mean township population 2005–2018 = 17,602 people; mean township area = 144 km^2^) were calculated by combining the predicted NO_2_ concentrations with 100-m gridded annual population estimates [39,40].

## 3. Results

### 3.1. NO_2_ Measurements

Mean annual NO_2_ concentrations at model development sites (n = 105) ranged from 7 to 49 ppb, with an overall mean of 27.6 ppb (SD = 10 ppb) (Figure S5).

### 3.2. Model Performance

The final LUR model is shown in Table 2. Overall, the model captured 63% of annual NO_2_ in NHAR (RMSE: 6 ppb (21% of the mean of all monitoring sites)) in 2019. The satellite-derived estimate of tropospheric NO_2_ accounted for the large majority of prediction performance of the model (incremental adjusted R^2^ = 45%)_,_ while the major roads (8%), vegetation (8%), and impervious surfaces variables (2%) made more modest contributions.

### 3.3. Model Diagnostics and Cross-Validation

There was no evidence of autocorrelation or other violations of linear regression assumptions in model residuals (Figures S1 and S4). VIF values did not suggest collinearity among the predictors (Table 2). Cook’s distance and df-beta plots identified four sites with pronounced influence on model predictions and coefficients. Initially, each of these monitoring locations were checked for errors in input data and manually searched to assess the nature of the sites and value of retaining them in the data. Additionally, they were sequentially removed and their effect on model parameters and output evaluated (predictors selected, coefficients, significance values of included variables, and adjusted R^2^). Ultimately, all four were excluded, leaving 105 sites. Minimal changes to model output metrics (RMSE, R^2^, and MAE) were observed when the cross-validation process was repeated 50 (RMSE: 6.1; R^2^: 0.63; MAE: 4.9) and 500 times (RMSE: 6.1; R^2^: 0.64; MAE: 4.9) (Table S3).

### 3.4. Historical Validation

Results of independent evaluation performed using historical NO_2_ measurements from monitoring sites not included (n = 41) in model development are shown in Table S5. When the final 2019 LUR model coefficients were applied to the corresponding predictor values for the 16 new sites from year 2014 and additional 25 monitoring locations from 2015, a reduction of −0.09 to −0.10 in R^2^ (RMSE: 4.9 ppb) was observed. Based on the mean bias (MB) values, our LUR model on average underpredicts historic NO_2_ concentrations by −0.5 to −0.6 ppb when exposed to new data (Figure S6).

### 3.5. Model Predictions

**Figure 2:**
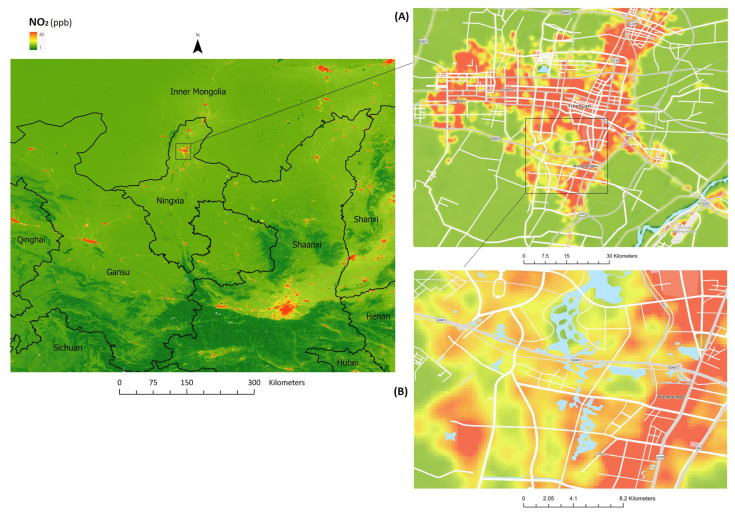
Mean annual NO_2_ model predictions for Ningxia and surroundings (2019), gridded at 100 m (black polygon lines represent provincial level divisions in the region). Inset (**A**) is of Yinchuan (2.3 million population), capital of Ningxia Province (7.2 million population). Inset (**B**) highlights local variability in NO_2_ concentrations in urban locations in Yinchuan (Estimated range: 12–38 ppb) (white polylines in insets (**A**,**B**) represent major roads).

Model predictions for annual NO_2_ concentrations for Ningxia and surrounds are displayed in Figure 2. In less densely populated and more remote areas in the south-western parts of the region, the model estimated NO_2_ concentrations ranging from 4 to 6 ppb.

Inset A of Figure 2 focuses on Yinchuan (capital of NHAR; population: 2.3 million). Maximum NO_2_ levels (40 ppb) were observed in congested areas along major roads as well as industrial and commercial districts. This pattern was consistent in other urban areas, such as Xi’an (Shaanxi Province), Lanzhou (Gansu Province), Batou (Inner Mongolia), and Hohhot (Inner Mongolia). Historic predictions (2005–2018) for 358 township-level divisions in Ningxia province are shown in Table 3. Overall, a slightly increasing trend in estimated population-weighted annual average concentrations was observed.

## 4. Discussion

### 4.1. Overall Findings and Model Performance

The model captured 63% of annual NO_2_ in NHAR (RMSE: 6 ppb (21% of the mean of all monitoring sites) in 2019 (Table 2). When evaluated for prediction performance and error using independent sites from 2014 and 2015 (n = 41 different sites), a reduction of −0.09 to −0.10 in R^2^ (RMSE: 4.9 ppb) was observed, which was consistent with other comparative LUR models in the literature (Table S5) [7,20,21,23,26,27,41,42]. As the increases in estimates noted for township-level divisions in NHAR (2005–2018) are minimal (0.2 ppb annual average increase) and within the prediction error of the model (RMSE: 6.5 ppb) and historical validation (2014–2015) (RMSE: 4.9 ppb), there was no clear evidence of an increasing trend.

### 4.2. Comparison with Existing LUR Models in China

The relatively low adjusted R2 value (63%) of our model highlights the challenges of capturing the diverse range of mobile and static sources of a highly spatially heterogenous pollutant, such as NO_2_. However, the parsimonious nature of the model (four predictors) may also explain why a modest 9–10% reduction in R^2^ (RMSE: 4.9 ppb) from training to external validation was observed, suggesting robustness when predicting at locations beyond the training sites, as is done in epidemiological studies. However, the spatial variation in NO_2_ concentrations explained by the LUR model is within the range of R^2^ values (0.51–0.78) reported for previous LUR models for NO_2_ estimation in China [7,20,21,23,26,27,41,42]. Of the eight comparable annual LUR models (five city-level models, R^2^: 0.51–0.67; two regional models, R^2^: 0.51–0.67; and one national model, R^2^: 0.78), only two included satellite observations of NO_2_ in model development [7,23]. In these models (regional model = R^2^: 0.61; national model = R^2^: 0.78), tropospheric NO_2_ measurements improved model performance by 12 to 13.5 percentage points [7,23]. In our LUR model, which has been developed for a less densely populated region, satellite measurements alone accounted for 45% of prediction of NO_2_ levels. We assume that the satellite observations captured important regional-scale emissions from other provinces that affected local variations in NO_2_ in Ningxia.

The reported differences in R^2^ values among annual LUR models for NO_2_ in China can be attributed to variation in the availability of data to inform the models. In particular, the distribution and density of monitoring sites, the availability of high-resolution data on land cover, and the use of satellite-based observations of NO_2_ are important factors that differ among the reported models [7,19]. Furthermore, regional and city-specific models explored smaller buffer radii around each monitoring site (0.3 to 3 km) compared to our model (0.1 to 10 km), which may have affected overall performance [20,21,22,23,26,41]. Improved performance was only noted in one national-level model (78%). Xu and colleagues included ground-level measurement data from China’s expanding national monitoring network consisting of more than 1382 sites [7]. The number and density of sites used likely increased their model’s ability to capture fine-scale distribution patterns of NO_2_ [7].

Despite methodological differences between our LUR model and existing regional and national models, some similarities were observed. For instance, predictors of traffic-related NO_2_ pollution (major road length) were included in all models since vehicle emissions are a major source of the pollutant. Similarly, the inclusion of predictors of man-made or impervious surfaces were also prominent in most models, as they account for anthropogenic activities. Likewise, vegetation cover, which is negatively associated with ambient NO_2_ concentration, was commonly featured in existing LUR models [7,20,21,23,24,25,26,27,28,41,42]. Industrial land use was not chosen by the forward stepwise regression process in our model. Considering the remoteness of the region, there are generally fewer industrial sources of NO_2_ than in larger metropolitan centres and their surrounding peri-urban areas in eastern China. The study domain also had a lower density of coal power stations (~3 per 10,000 km^2^) compared to more populated regions in China (~9 per 10,000 km^2^) [32]. However, tropospheric OMI observations, which explained 45% of the spatial variability of the pollutant in the study area, likely captured industrial contributions to NO_2_ emissions, in addition to other sources.

### 4.3. Limitations

There was evidence of underestimation of historical NO_2_ concentrations by 0.5 to 0.6 ppb (~22%). However, underestimation of NO_2_ levels is more likely at higher values rather than across the range of NO_2_ predictions (Figure S7). LUR models are also known to be more reliable in estimating mean exposures than extreme values of NO_2_ (Xu et al. 2019).

When evaluated using independent sites from 2014 and 2015, which is a more rigorous and realistic test of a model’s performance (including historical performance up to five years prior), a drop of −0.09 to −0.10 in R^2^ was observed (Table S5). The RMSE of 4.9 ppb obtained for historic validation (2014–2015) and for the 2019 model (RMSE: 6.1 ppb) suggest that the level of uncertainty or error in predictions for 2005–2019 is likely in the range of 4.9 to 6.5 ppb. This decrement in performance and in general RMSE error range is consistent with other LUR models [16]. Possible explanations include the fact that major road length and impervious surface cover were not available historically. However, as tropospheric satellite observations of NO_2_ (data dating back to 2004) accounted for most of the prediction performance, it provided confidence in applying our model to earlier years.

Additional sources of error may include the use of open-source ground-level monitoring data, which may have been incomplete. As the data were not obtained directly from environmental monitoring agencies in China, sites measuring important local sources of NO_2_ may have been missed. The locations of air quality monitors in the study area (primarily near government institutions, including schools, universities, and recreational areas) may have limited their ability to capture important spatial gradients relating to industry and airports. Model performance may also be overly optimistic, as we were unable to test our LUR model against more historic ground-level monitoring measurements (apart from 2014 and 2015) due to unavailability of data for the region.

## 5. Conclusions

The finding that 45% of NO_2_ was captured by satellite-derived estimates of tropospheric NO_2_ alone, which date back to 2004, along with the historical validation we undertook, provides support for applying 2019 coefficients to earlier years. The traditional LUR approach we used, which emphasised empirical plausibility and interpretability of coefficients, offered modestly improved predictions (2–4% improvement in R^2^) compared with other sub-national models for China. More importantly, the model yields valid estimates of annual NO_2_ in Ningxia and its immediate surrounding areas, and it will be used to estimate NO_2_ exposure within that spatial domain in an epidemiological study.

## Figures and Tables

**Table 1:** Predictor variables included in LUR model development *.

Variable (Units)	Spatial Resolution	Point or Buffer Estimate
OMI (Ozone Monitoring Instrument) NO_2_ observations (ppb)	10 km	Point
Elevation (m)	90 m	Point
Annual mean Temperature (°C)	1 km	Point
Annual mean Precipitation (mm)	1 km	Point
Distance to nearest major road (km)	-	Point
Distance to nearest coal power station (km)	-	Point
Vegetation cover (%)	250 m	Buffer Average
Tree cover (%)	30 m	Buffer Average
Impervious surfaces (%)	250 m	Buffer Average
Water cover (%)	500 m	Buffer Average
Active Fires (fires/1000 km^2^/day)	10 km	Buffer Sum
Population density (persons/km^2^)	1 km	Buffer Average
Major roads (km)	-	Buffer Sum
Minor roads (km)	-	Buffer Sum
Power Plant Emissions (tons of CO_2_/year)	-	Buffer Sum
Land use by type—Residential, Commercial, and Industrial (%)	-	Buffer Average

* Further infor mation on predictor data sources can be found in Table S2. Buffer estimates were obtained for 22 buffer sizes, ranging from 100 m, 200 m, 300 m, 400 m, 500 m, 600 m, 700 m, 800 m, 1000 m, 1200 m, 1500 m, 1800 m, 2000 m, 2500 m, 3000 m, 3500 m, 4000 m, 5000 m, 6000 m, 7000 m, 8000 m, and 10,000 m.

**Table 2:** Summary of final LUR Model.

Final Model Output	Predictor, Buffer (Units)	*Β* *	SE	Adj. R^2^	VIF	Contribution to Model (%)
R^2^: 0.64	Intercept	27.40	0.59			
Adj. R^2^: 0.63	(OMI) tropospheric NO_2_, (ppb)	6.03	0.83	0.45	1.96	45%
RMSE: 6.1 ppb	Major roads, 5 km (km)	3.02	0.80	0.53	1.76	8%
% RMSE: 21.9 %	Vegetation cover, 1.8 km (%)	−3.43	0.71	0.61	1.47	8%
	Impervious surface, 7 km (%)	1.87	0.76	0.63	1.67	2%

* All predictors, including intercept, were significant at <0.05. Predictors were standardised and mean centred to allow for better interpretation of coefficients (see supplementary information). Predictors are listed in the order they were added to the model. SE, standard error; VIF, variance inflation factor; RMSE, root mean squared error (expressed as absolute in ppb and % of mean NO_2_ for all sites); ppb, parts per billion.

**Table 3:** Selected percentiles of NO_2_ concentrations (ppb) predicted for 358 township-level divisions in Ningxia Province (2005–2018).

Year	5th	25th	50th	75th	95th	Unweighted Average	Population Weighted Average
2005	11.0	13.6	14.5	17.4	21.7	14.9	15.6
2006	11.1	14.0	14.7	17.6	21.9	15.4	16.1
2007	11.2	14.1	15.0	17.9	22.0	15.6	16.3
2008	11.4	14.3	15.1	17.9	22.2	15.7	16.4
2009	11.5	14.3	15.1	18.0	22.4	15.9	16.6
2010	11.6	14.3	15.3	18.0	22.6	15.9	16.6
2011	11.8	14.4	15.5	18.0	22.8	16.1	16.7
2012	12.0	14.5	15.6	18.1	23.0	16.5	17.0
2013	12.2	14.7	15.6	18.2	23.1	16.7	17.2
2014	12.3	14.8	15.7	18.4	23.3	16.8	17.3
2015	12.4	14.8	15.8	18.6	23.5	16.7	17.3
2016	12.5	14.8	15.8	18.7	23.7	16.8	17.4
2017	12.7	15.0	15.9	18.7	23.7	16.9	17.5
2018	12.8	15.2	16.1	18.7	23.8	16.9	17.5

## Data Availability

Data is contained within the article and supplementary material. The data presented and used in this study are available in [NO_2_ LUR Model Manuscript—Supplementary Information].

## Supplementary Materials

The following are available online at https://www.mdpi.com/article/10.3390/ijerph182412887/s1, NO_2_ LUR Model Manuscript—Supplementary Information. Figure S1: Distribution of final LUR model residuals, Figure S2: Cook’s distance plot for all sites included in final LUR model, Figure S3 (A): DF-BETA statistics plots for each predictor included in final LUR model. * OMINO2 represents tropospheric NO_2_ measurements. MR5000, VC1800 & IS7000 stand for major roads (5 km), vegetation cover (1.8 km) and impervious surfaces (7 km), Figure S3 (B): DF-BETA statistics plots for each predictor included in final LUR model. * MR5000, VC1800 & IS7000 stand for major roads (5 km) and vegetation cover (1.8 km), Figure S3 (C). DF-BETA statistics plots for each predictor included in final LUR model. * IS7000 stands for impervious surfaces (7 km), Figure S4: Global Moran’s I Summary for final LUR model residuals, Figure S5: Distribution of mean annual hourly average NO_2_ (ppb) concentrations observed at 123 monitoring sites in Ningxia and surrounding areas in 2019, Figure S6: Bland-Altman Plot showing the level of agreement between predicted NO_2_ concentrations (using coefficients from 2019 LUR model applied to time-varying predictors) and actual concentrations measured at historical sites (2014 & 2015) not used in model development (n = 41), Figure S7: Predicted vs. observed annual mean NO_2_ (ppb) concentrations (2019) with a 1:1 regression line, Table S1: Location and number of monitoring sites by province (year 2019), Table S2: Data sources of predictor variables, Table S3: Results of k-fold cross-validation (5-fold cross-validation, Table S4: Descriptive statistics of annual mean NO_2_ (ppb) at model evaluation sites for years 2014 and 2015, Table S5: Independent Evaluation of LUR model at 41 Monitoring sites (Measured NO_2_ regressed on Predicted NO_2_ using LUR model).
