# Supplementary material for: Development and Validation of a Sub-National, Satellite-Based Land-Use Regression Model for Annual Nitrogen Dioxide Concentrations in North-Western China
Source: Int J Environ Res Public Health. 2021 Dec 7;18(24):12887. doi: 10.3390/ijerph182412887 (PMC8701972; doi:10.3390/ijerph182412887)
Supplement: Supplementary file 1 [file ijerph-18-12887-s001.zip › ijerph-1438520-supplementary.pdf]

# Development and validation of a sub-national, satellite-based land-use regression model for annual nitrogen dioxide concentrations in North-Western China

**Igor Popovic[1,2*], Ricardo J. Soares Magalhães[2,3], ShuKun Yang[4], YuRong Yang[5], Erjia Ge[6], Boyi Yang[7], Guang-Hui Dong[8], Xiaolin Wei[6], Guy B. Marks[9,10,11], Luke D. Knibbs[11,12]**

**Affiliations:**

1. Faculty of Medicine, School of Public Health, University of Queensland, Herston, 4006, Australia;

2. UQ Spatial Epidemiology Laboratory, School of Veterinary Science, University of Queensland, Gatton, 4343, Australia;

3. Children's Health and Environment Program, UQ Children's Health Research Center, The University of Queensland, South Brisbane, 4101, Australia;

4. Department of Radiology, The Second Affiliated Hospital of Ningxia Medical University, The First People's Hospital in Yinchuan, Yinchuan, 750000, China;

5. Department of Pathogenic Biology & Medical Immunology, School of Basic Medical Science, Ningxia Medical University, Yinchuan, 750000, China;

6. Dalla Lana School of Public Health, University of Toronto, Toronto, M5S 1A1, Canada

7. Guangdong Provincial Engineering Technology Research Center of Environmental Pollution and Health Risk Assessment, Department of Occupational and Environmental Health, School of Public Health, Sun Yat-sen University, Guangzhou, 510085, China;

8. Guangzhou Key Laboratory of Environmental Pollution and Health Risk Assessment, Department of Preventive Medicine, School of Public Health, Sun Yat-sen University, Guangzhou, 510085, China;

9. South Western Sydney Clinical School, University of New South Wales, Liverpool, 2170, Australia;

10. Woolcock Institute of Medical Research, Glebe, 2037, Australia;

11. Centre for Air Pollution, Energy and Health Research, Glebe, 2037, Australia;

12. Faculty of Medicine and Health, School of Public Health, The University of Sydney, Camperdown, 2006, Australia.

**[*] Corresponding author:**

Igor Popovic, School of Public Health, Faculty of Medicine, University of Queensland, Herston, Australia.

E-mail address: i.popovic@uq.edu.au

**CONTENTS**

**1 Methods** S3

1.1 Satellite retrieval of nitrogen dioxide…….……………………………………..S3

1.2 Land use data..………………………………………………….....................S3

1.3 Spatial covariates..……………………………………………………………… S4

1.4 Monitoring sites….………………………………………………………………S5

**2 Results** S8

2.1 Model checking……….….……………………………………………………...S8

2.2 Measured $NO_2$ concentrations………………...………………………………….S14

2.3 Cross-validation.……………………………………….....………………… S14

2.4 Independent evaluation….……………………………………………………….S14

**References**………………………………………………………………………….S19

## 1. METHODS

### 1.1 Satellite Retrieval of Nitrogen Dioxide

To supplement predictors used in this model as well as areas in the study region that lack coverage by ground-based monitoring stations, monthly mean tropospheric $NO_2$ measurements were retrieved from the NASA Ozone Monitoring Instrument (OMI) and aggregated to annual averages [1, 2]. OMI was launched on July 15, 2004, on the NASA Aura satellite, which is in a sun-synchronous ascending polar orbit with a local equator crossing time (LECT) of 13:45±0:15. The OMI algorithm relies on estimated column concentrations (total, tropospheric and stratospheric) of $NO_2$ from daily solar spectral irradiance measurements taken by the Ozone Monitoring Instrument. The radiance values are subject to radiometric, de-striping as well as satellite path slant corrections and air mass factors and are subsequently converted from satellite swatch grid pixels (13 x 24 km at nadir) to a consistent grid size [1, 2]. The OMI $NO_2$ (Collection 3.0, Version 4.0) product used in our model is a high spatial resolution (0.1°) version of the OMNO2d standard level 3 product available at 0.25 x 0.25 degrees with units of $10^{15}$molecules/cm$^2$. This version of the Aura Ozone Monitoring Instrument (OMI) also includes a number of updates: (1) use of a new daily and OMI field of view specific geometry dependent surface Lambertian Equivalent Reflectivity (GLER) product in $NO_2$ and cloud retrievals; (2) improved cloud parameters using a new $O_2$-$O_2$ slant column data and the GLER product for terrain reflectivity; (3) more accurate terrain pressure calculated using OMI ground pixel-averaged terrain height and monthly mean GMI terrain pressure; and (4) improved treatment over snow/ice surfaces by using the concept of scene LER and scene pressure [1, 2].

### 1.2 Land Use

Land use data was sourced from Geofabrik servers, with daily updated data extracts originating from the OpenStreetMap Project (Table S1) [3]. China specific ESRI shapefiles classifying land use into residential, commercial and industrial land according to the Chinese Land Use Classification Standard (GB/T 21010-2007) were analysed using ArcGIS Pro 2.7. Similarly, satellite-derived predictors including vegetation, tree and water cover, as well as impervious surface extent and active fires (fires/1000km$^2$/day) were also incorporated in model development [4-8].

### 1.3 Spatial Covariates

Gridded Population of the World (GPW version 4) data estimates of population density (number of persons per square km) were obtained from the Centre of International Earth Science Information Network (CIESIN – Columbia University) [9]. Estimations assign population counts using sub-national administrative units to 30 arc-second grid cells which are consistent with national censuses and population registers. The spatial resolution of each cell is the finest resolution global population density estimate data currently available (Table S1).

Road intensity (major and minor roads) data was sourced from OpenStreetMap (OSM) [3]. Total road length was computed in ArcGIS Pro 2.7 to determine road intensity. Roads classified as major roads included motorways, trunk ways, primary roads connecting towns or main roads within metropolitan areas. Minor roads included small residential roads, pedestrian walkways which serve as access points to houses or residential complexes. Additionally, the distance to the nearest major road from each monitoring site was included for analysis as the inverse distance. Coal power plant emissions data including coordinates of each plant was compiled from Carbon Brief, a global power plant emissions inventory [10, 11]. Tons of $CO_2$ per year for each active plant was recorded in addition to distance to closest coal power plant. Predictors describing topographic (elevation) and meteorological (annual mean precipitation and temperature) characteristics at monitoring site locations were also collated and aggregated to annual averages (Table S2).

**1.4 Monitoring sites**

**Table S1**

Location and number of monitoring sites by province (year 2019).

| | ***n* monitors** | **TOTAL** |
|---|---|---|
| **Within Ningxia Province** | | 19 |
| *Yinchuan Prefecture* | 6 | |
| *Zhongwei Prefecture* | 3 | |
| *Wuzhong Prefecture* | 3 | |
| *Shizuishan Prefecture* | 4 | |
| *Guyuan Prefecture* | 3 | |
| **500 km radius around Ningxia** | | 104 |
| *Inner Mongolia Province* | 28 | |
| *Gansu Province* | 28 | |
| *Shaanxi Province* | 48 | |
| | | **123** |

**Table S2**
Data sources of predictor variables.

| Variable (units) / expected effect direction (+ve/-ve) | Spatial Resolution | Point or Buffer Estimate | *Data Source (Year) / Web Link |
|---|---|---|---|
| Annual mean OMI tropospheric $NO_2$ (ppb) / +ve | 10 km | Point | GES DISC Earthdata – NASA (2019) / https://avdc.gsfc.nasa.gov/pub/data/satellite/Aura/OMI/V03/L3/OMNO2d_HR/OMNO2d_HRM/ |
| Elevation (m) / -ve | 90 m | Point | CGIAR-CSI GeoPortal (2018) / https://cgiarcsi.community/data/srtm-90m-digital-elevation-database-v4-1/ |
| Annual mean Temperature (°C) / +ve | 1 km | Point | WorldClim - University of East Anglia (2019) / https://www.worldclim.org/data/worldclim21.html |
| Annual mean Precipitation (mm) / -ve | 1 km | Point | WorldClim - University of East Anglia (2019) / https://www.worldclim.org/data/worldclim21.html |
| Inverse Distance to nearest major road (km) / +ve | - | Point | OpenStreetMap Data Extracts (2019) / https://download.geofabrik.de/asia/china.html |
| Inverse Distance to nearest coal power station (km) / +ve | - | Point | OpenStreetMap Data Extracts (2019) / https://download.geofabrik.de/asia/china.html |
| Vegetation cover (%) / -ve | 250 m | [a] Buffer Average | NASA Earth Observations MODIS/Terra Vegetation Continuous Fields Yearly (2019) / https://lpdaac.usgs.gov/products/mod44bv006/ |
| Tree cover (%) / -ve | 30 m | [a] Buffer Average | NASA Earth Observations GFCC30TC Tree Cover Multi-Year Global (2019) / https://lpdaac.usgs.gov/products/gfcc30tcv003/ |
| Impervious surfaces (%) / +ve | 250 m | [a] Buffer Average | SEDAC - Columbia University (2010) / https://sedac.ciesin.columbia.edu/data/set/ulandsat-gmis-v1 |
| Water cover (%) / -ve | 500 m | [a] Buffer Average | NASA Earth Observations MODIS/Terra+Aqua Land Cover Type Yearly (2019) / https://lpdaac.usgs.gov/products/mcd12q1v006/ |
| Active Fires (fires/1000 $km^2$/day) / +ve | 10 km | [b] Buffer Sum | NASA Earth Observations Active Fires Terra/MODIS (2019) / https://neo.sci.gsfc.nasa.gov/view.php?datasetId=MOD14A1_M_FIRE |
| Population density (persons/$km^2$) / +ve | 1 km | [a] Buffer Average | SEDAC - Columbia University (2019) / https://sedac.ciesin.columbia.edu/data/set/gpw-v4-population-density-rev11 |
| Major roads (km) / +ve | - | [b c] Buffer Sum | OpenStreetMap Data Extracts (2019) / https://download.geofabrik.de/asia/china.html |
| Minor roads (km) / +ve | - | [b d] Buffer Sum | OpenStreetMap Data Extracts (2019) / https://download.geofabrik.de/asia/china.html |
| Power Plant Emissions (tons of $CO_2$/year) / +ve | - | [b] Buffer Sum | Carbon Brief (2019) / https://www.carbonbrief.org/mapped-worlds-coal-power-plants |
| Land use by type – Residential, Commercial & Industrial (%) / +ve | - | [a] Buffer Average | OpenStreetMap Data Extracts (2019) / https://download.geofabrik.de/asia/china.html |

**NOTES:**

* Data sources were accessed August - September 2020.
[a] Average of variable within buffer were obtained for 22 buffer sizes ranging from 100m, 200m, 300m, 400m, 500m, 600m, 700m, 800m, 1000m, 1200m, 1500m, 1800m, 2000m, 2500m, 3000m, 3500m, 4000m, 5000m, 6000m, 7000m, 8000m & 10, 000m.
[b] Sum of variable within buffer for 22 different buffer radii (see above).
[c] Roads classified as major roads included motorways, trunk ways, primary roads connecting towns or main roads within metropolitan areas.
[d] Minor roads were defined as residential roads, pedestrian walkways which serve as access points to houses or residential complexes.

## 2. RESULTS

### 2.1 Model checking

Figures S1 – S4 show model residuals, Cook's distance and boxplots of df-beta statistics for each variable in the final LUR model and Global Moran's I summary for final LUR model residuals.

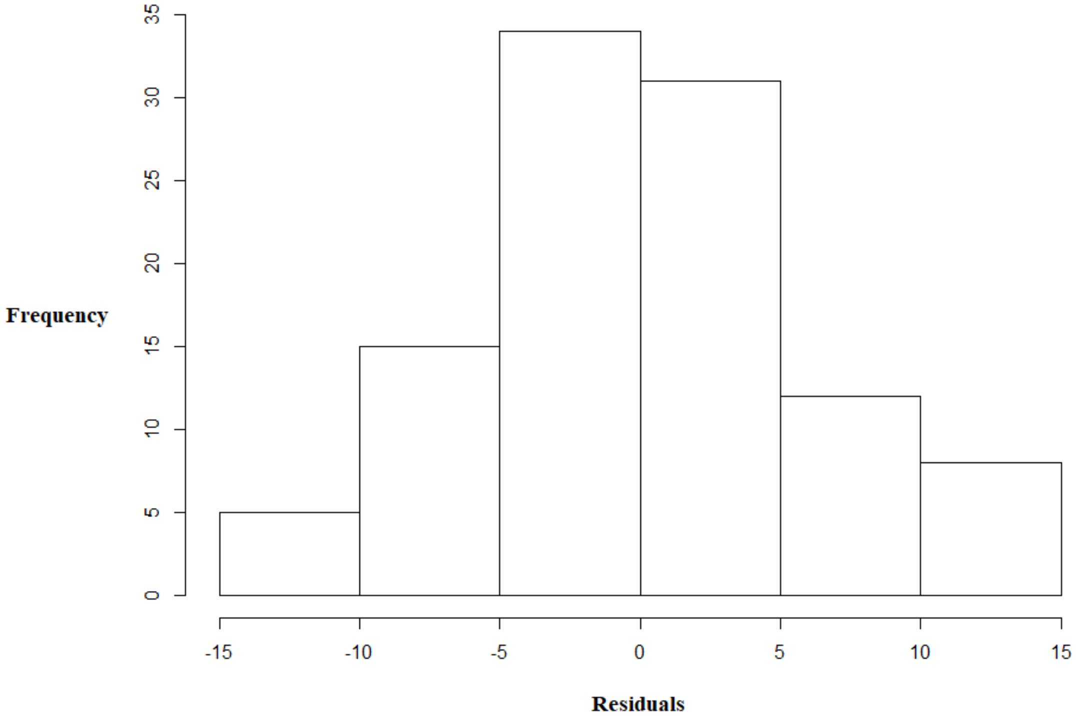


**Figure S1.** Distribution of final LUR model residuals.

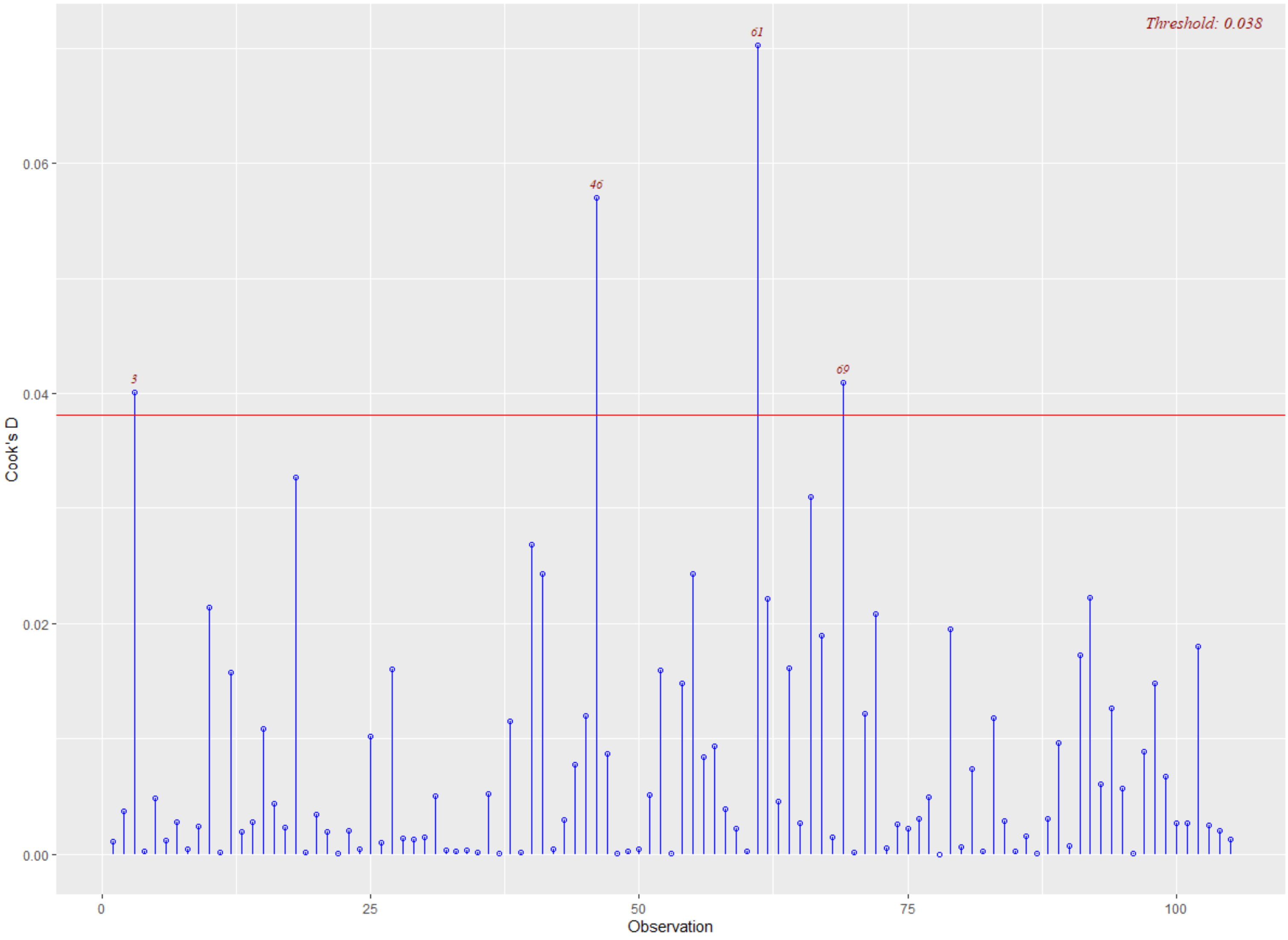


**Figure S2.** Cook's distance plot for all sites included in final LUR model.

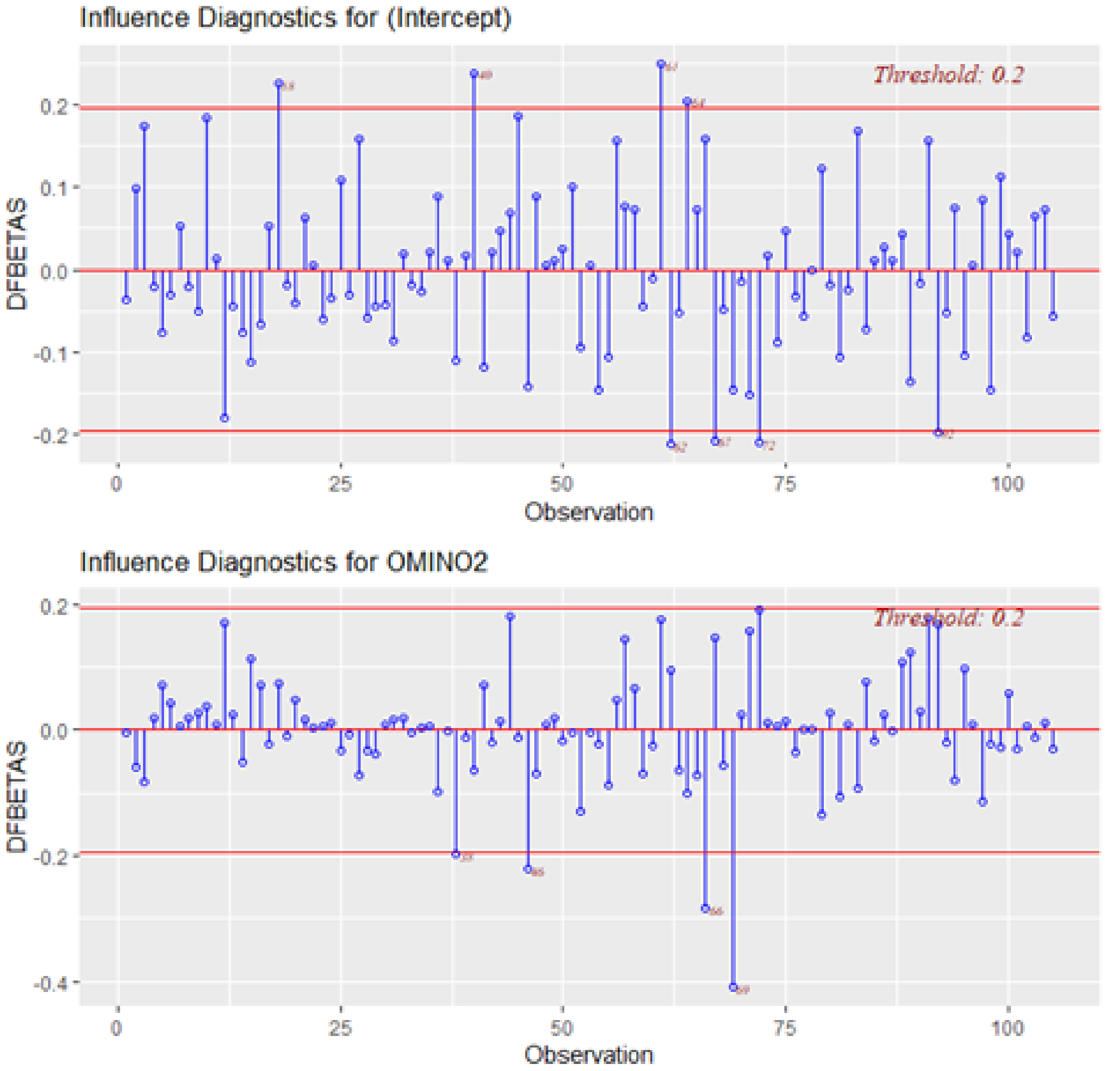


**Figure S3 (A).** DF-BETA statistics plots for each predictor included in final LUR model. *OMINO2 represents tropospheric $NO_2$ measurements. MR5000, VC1800 & IS7000 stand for major roads (5km), vegetation cover (1.8km) and impervious surfaces (7km).

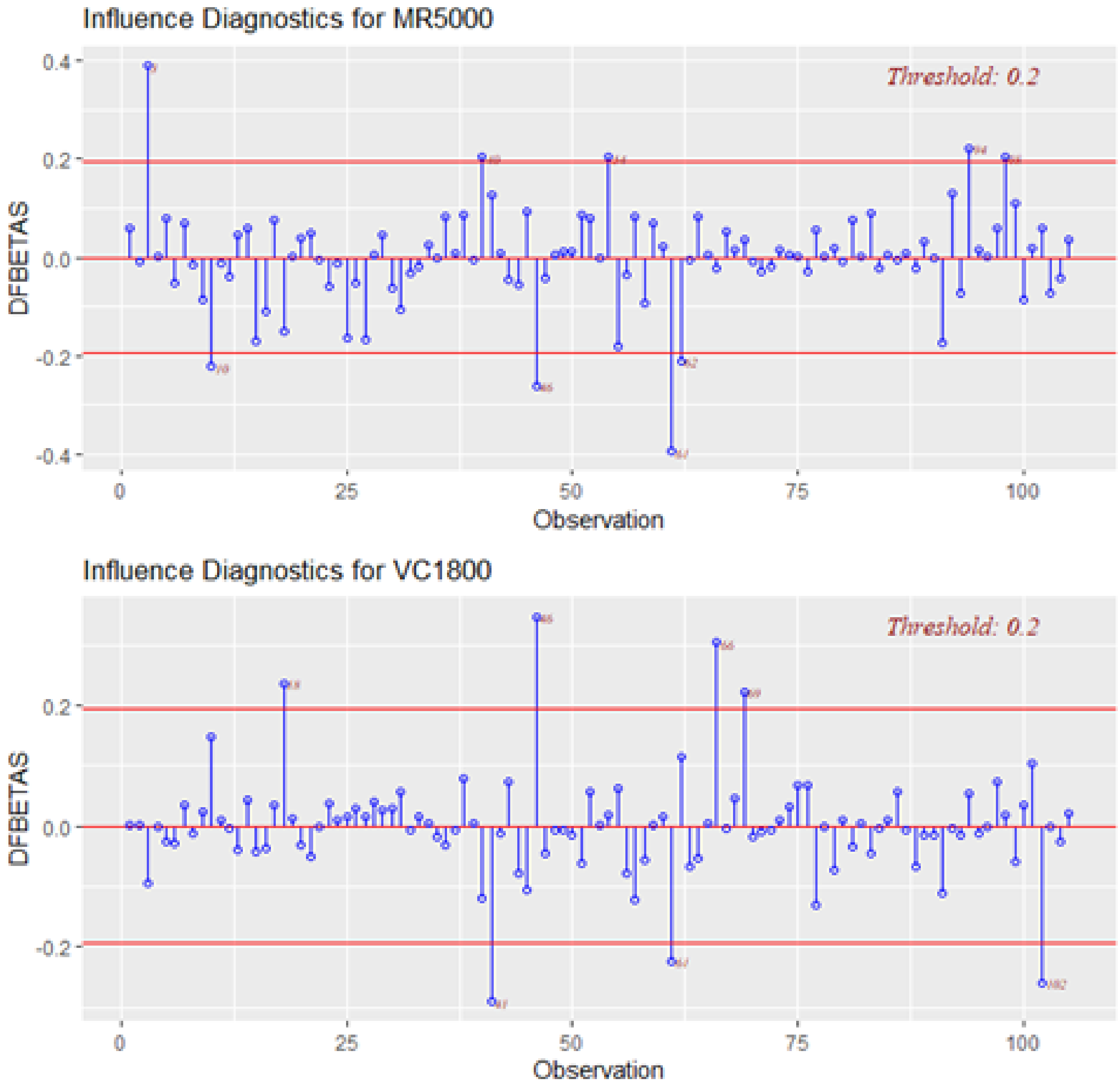


**Figure S3 (B).** DF-BETA statistics plots for each predictor included in final LUR model. * MR5000, VC1800 & IS7000 stand for major roads (5km) and vegetation cover (1.8km).

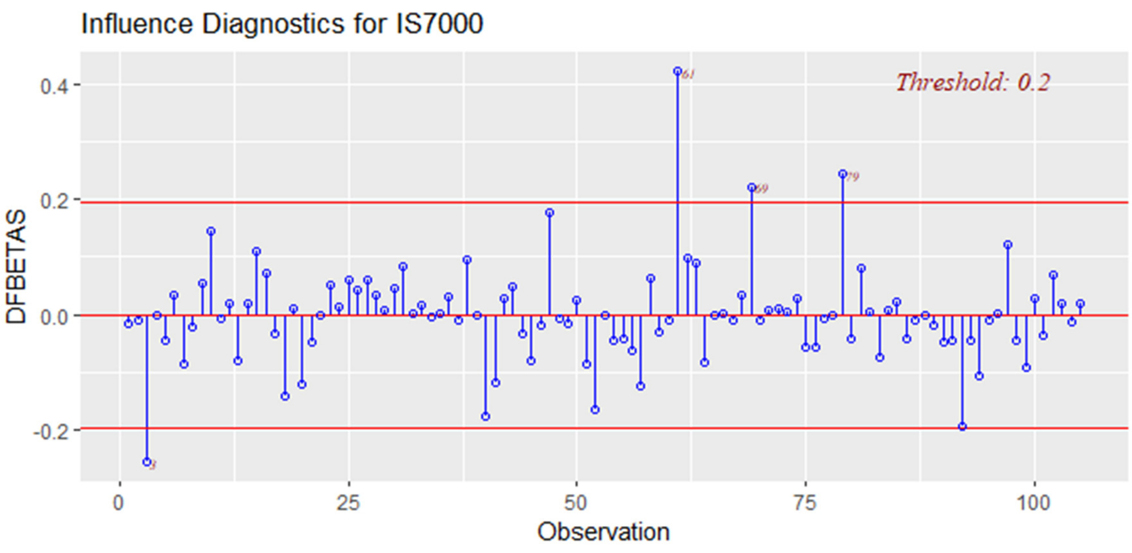


**Figure S3 (C).** DF-BETA statistics plots for each predictor included in final LUR model. *IS7000 stands for impervious surfaces (7km).

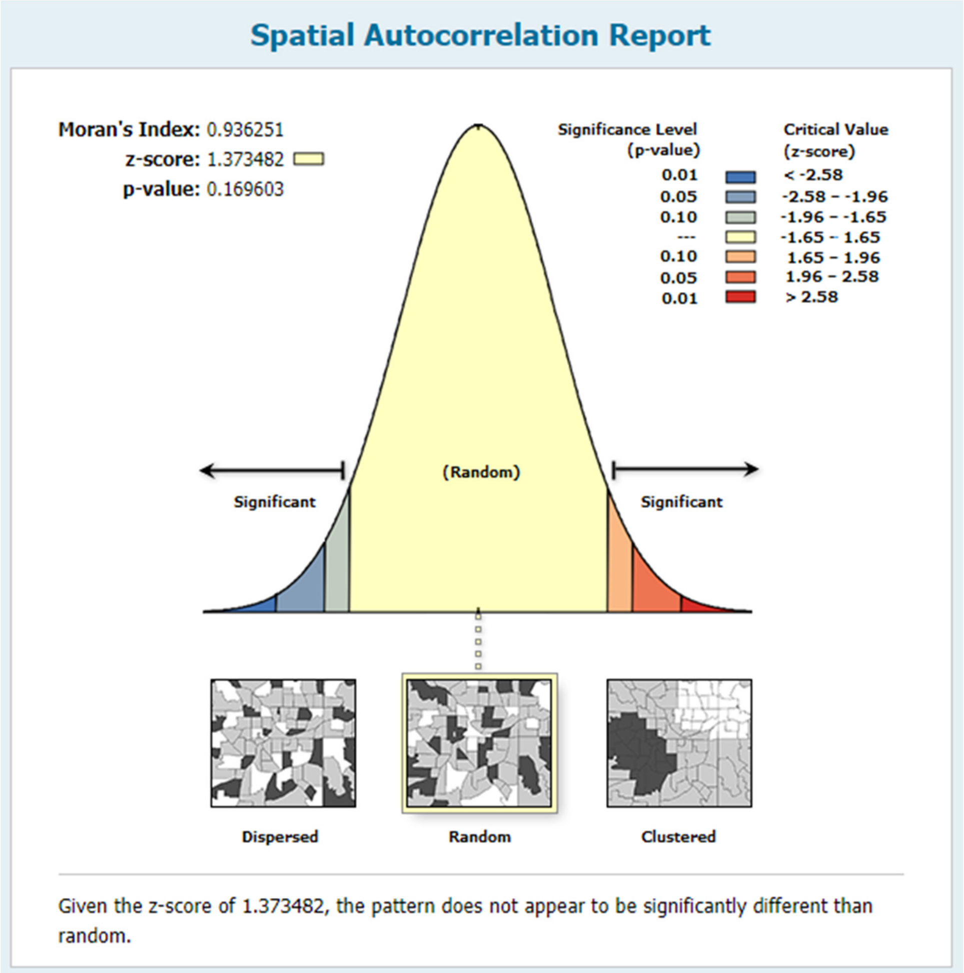


## Global Moran's I Summary

| | |
|---|---|
| **Moran's Index:** | 0.936251 |
| **Expected Index:** | -0.009615 |
| **Variance:** | 0.474256 |
| **z-score:** | 1.373482 |
| **p-value:** | 0.169603 |

**Figure S4.** Global Moran's I Summary for final LUR model residuals.

### 2.2 Measured $NO_2$ concentrations

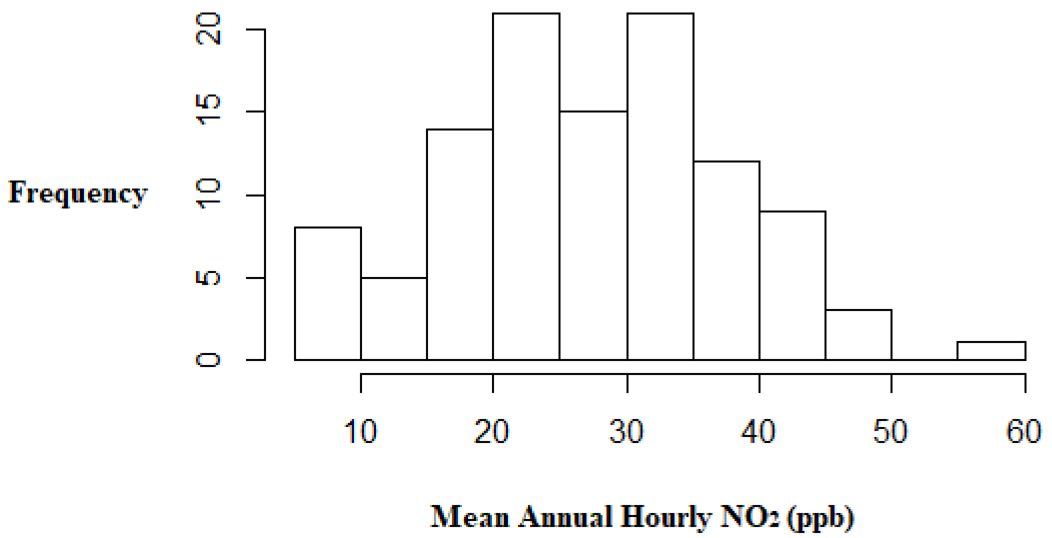


**Figure S5.** Distribution of mean annual hourly average $NO_2$ (ppb) concentrations observed at 123 monitoring sites in Ningxia and surrounding areas in 2019.

### 2.3 Cross-validation

The final LUR model was evaluated using k-fold cross-validation (5-fold cross-validation). Changes to model output metrics (RMSE, R2 & MAE) observed when the cross-validation process was repeated 50 and 500 times are shown in Table S4.

**Table S3**

Results of k-fold cross-validation (5-fold cross-validation).

| | 50 Repeats | 500 Repeats |
|---|---|---|
| **Adjusted $R^2$** | 0.62 | 0.62 |
| **$R^2$** | 0.63 | 0.64 |
| **RMSE** | 6.1 | 6.1 |
| **MAE** | 4.91 | 4.90 |

### 2.4 Independent Evaluation

Results of independent evaluation analyses using $NO_2$ measurements from monitoring sites not included (n = 41 different sites) in model development are shown in Table S5. We identified 41 additional sites (n = 16; year 2014 / n = 25; year 2015) for independent

evaluation that were not used for model development (Table S3). Our LUR model was used to predict annual average $NO_2$ concentrations for the period (2014 & 2015) that a site had valid data by applying the annual time-varying predictors to the same year. Results of independent evaluation are shown in Table S5.

**Table S4**

Descriptive statistics of annual mean $NO_2$ (ppb) at model evaluation sites for years 2014 and 2015.

| ***n*** | **Mean** | **SE** | **SD** | **Min** | **5th** | **25th** | **50th** | **75th** | **95th** | **Max** |
|---|---|---|---|---|---|---|---|---|---|---|
| 41 | 20.3 | 1.2 | 7.4 | 11.0 | 11.1 | 14.0 | 19.0 | 26.0 | 31.9 | 45.0 |

**Table S5**

Independent Evaluation of LUR model at 41 Monitoring sites (Measured $NO_2$ regressed on Predicted $NO_2$ using LUR model).

| | $R^2$ | $R^2$ change* | $\beta$ | SE | Intercept | RMSE | RMSE (%) | MSE | MB (ppb) | FB |
|---|---|---|---|---|---|---|---|---|---|---|
| Monitoring sites from year 2014, $n = 16$ | 0.54 | -0.10 | 0.58 | 0.14 | 8.07 | 4.9 | 22.47 | 24.45 | -0.61 | -0.0131 |
| Monitoring sites from year 2015, $n = 25$ | 0.55 | -0.09 | 0.55 | 0.07 | 5.29 | 4.9 | 22.22 | 23.94 | -0.57 | -0.0069 |

*Change from LUR model $R^2$; MSE = mean square error; SE = standard error; RMSE = root mean squared error (expressed as absolute and % of mean $NO_2$ for all sites); MB = mean bias; FB = fractional bias (dimensionless).

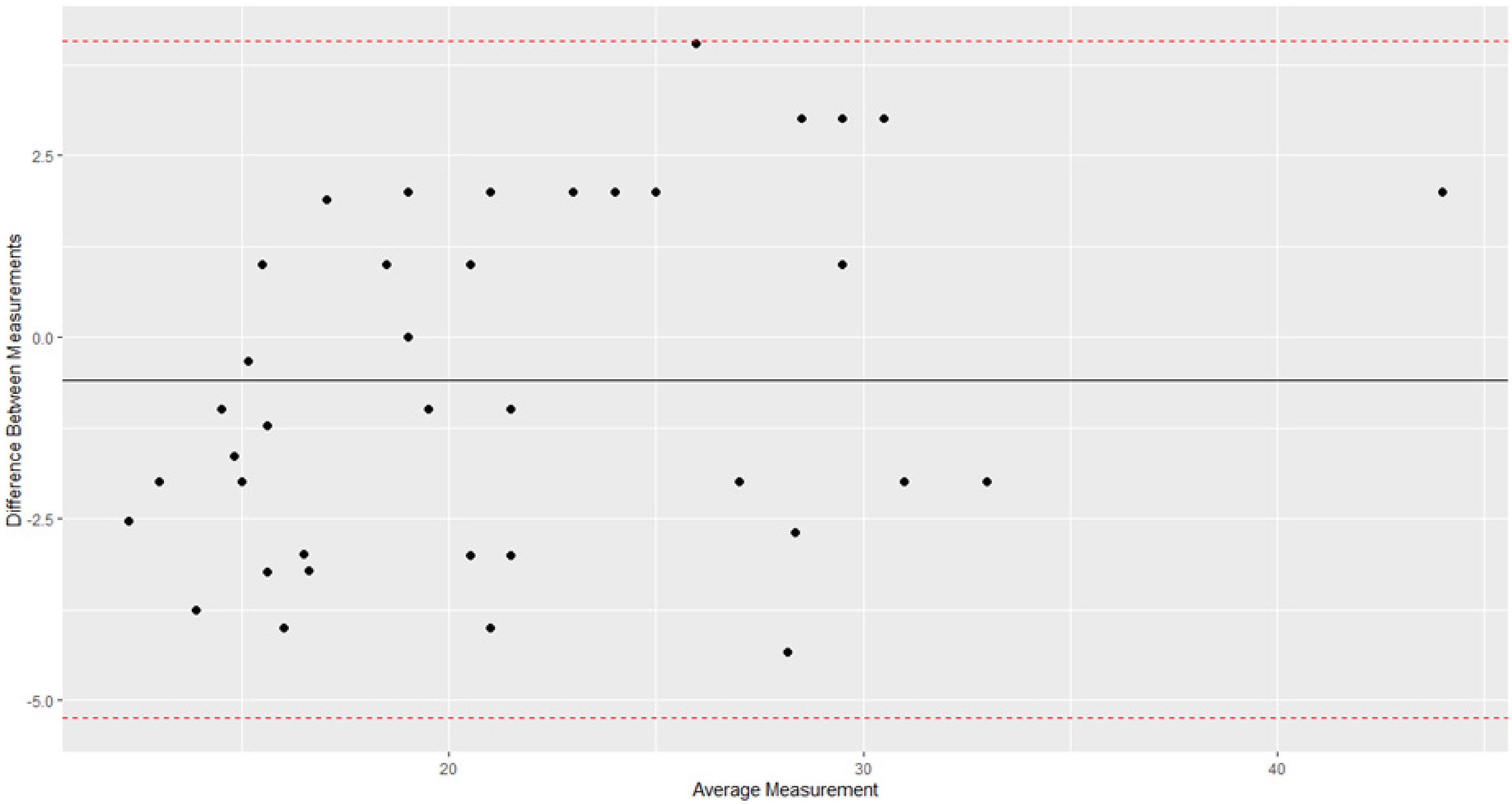


**Figure S6.** Bland-Altman Plot showing the level of agreement between predicted $NO_2$ concentrations (using coefficients from 2019 LUR model applied to time-varying predictors) and actual concentrations measured at historical sites (2014 & 2015) not used in model development (n = 41).

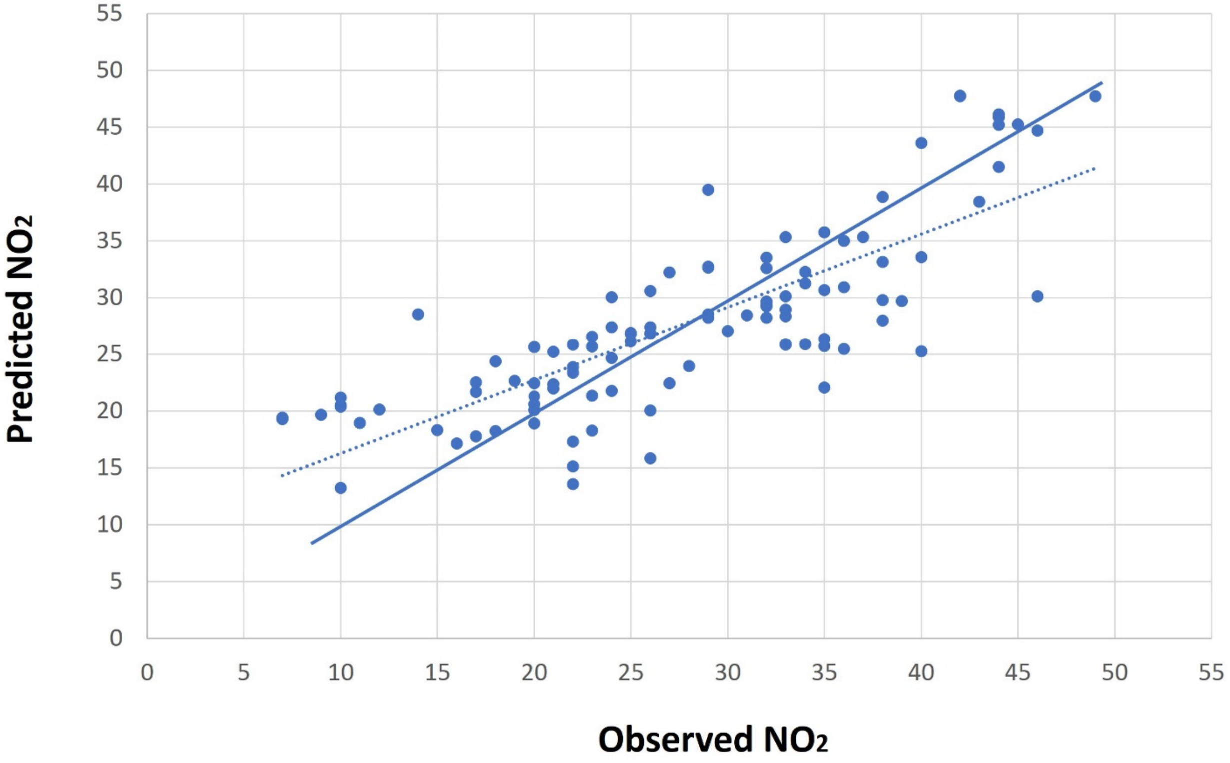


**Figure S7.** Predicted vs. observed annual mean $NO_2$ (ppb) concentrations (2019) with a 1:1 regression line.

## REFERENCES


1. Lamsal, L.N., et al., *Ozone Monitoring Instrument (OMI) Aura nitrogen dioxide standard product version 4.0 with improved surface and cloud treatments.* Atmos. Meas. Tech., 2021. **14**(1): p. 455-479.
2. Nickolay A. Krotkov, L.N.L., Sergey V. Marchenko, Eric J.Bucsela, William H. Swartz, Joanna Joiner and the OMI core team, *OMI/Aura Nitrogen Dioxide (NO2) Total and Tropospheric Column 1-orbit L2 Swath 13x24 km V003.* 2019.
3. Geofabrik, *OpenStreetMap Data China*. 2019.
4. Brown de Colstoun, E.C., C. Huang, P. Wang, J. C. Tilton, B. Tan, J. Phillips, S. Niemczura, P.-Y. Ling, and R. E. Wolfe, *Global Man-made Impervious Surface (GMIS) Dataset From Landsat*. 2017.
5. DiMiceli, C., Carroll, M., Sohlberg, R., Kim, D., Kelly, M., Townshend, J., *MOD44B MODIS/Terra Vegetation Continuous Fields Yearly L3 Global 250m SIN Grid V006*. 2019.
6. Friedl, M., Sulla-Menashe, D., *MCD12Q1 MODIS/Terra+Aqua Land Cover Type Yearly L3 Global 500m SIN Grid V006* 2019, NASA EOSDIS Land Processes DAAC.
7. NASA, *Active Fires (1 month - Terra/MODIS)*. 2019.
8. Townshend, J., *Global Forest Cover Change (GFCC) Tree Cover Multi-Year Global 30 m V003* 2019, NASA EOSDIS Land Processes DAAC.
9. SEDAC, *Gridded Population of the World, Version 4 (GPWv4): Population Density, Revision 11*. 2020, Center for International Earth Science Information Network - CIESIN - Columbia University.
10. CarbonBrief, *Global Coal Power Plant Emissions*. 2019.
11. Larkin, A., et al., *Global Land Use Regression Model for Nitrogen Dioxide Air Pollution.* Environ Sci Technol, 2017. **51**(12): p. 6957-6964.
